# Genetic Interactions Explain Variance in Cingulate Amyloid Burden: An AV-45 PET Genome-Wide Association and Interaction Study in the ADNI Cohort

**DOI:** 10.1155/2015/647389

**Published:** 2015-09-03

**Authors:** Jin Li, Qiushi Zhang, Feng Chen, Jingwen Yan, Sungeun Kim, Lei Wang, Weixing Feng, Andrew J. Saykin, Hong Liang, Li Shen

**Affiliations:** ^1^Institute of Biomedical Engineering, College of Automation, Harbin Engineering University, 145 Nantong Street, Harbin 150001, China; ^2^Center for Bioinformatics, College of Automation, Harbin Engineering University, 145 Nantong Street, Harbin 150001, China; ^3^College of Information Engineering, Northeast Dianli University, 169 Changchun Street, Jilin 132012, China; ^4^Center for Neuroimaging, Department of Radiology and Imaging Sciences, Indiana University School of Medicine, 355 West 16th Street, Suite 4100, Indianapolis, IN 46202, USA; ^5^Center for Computational Biology and Bioinformatics, Indiana University School of Medicine, 410 West 10th Street, Suite 5000, Indianapolis, IN 46202, USA; ^6^Department of Biohealth Informatics, Indiana University School of Informatics and Computing, Indianapolis, IN 46202, USA

## Abstract

Alzheimer's disease (AD) is the most common neurodegenerative disorder. Using discrete disease status as the phenotype and computing statistics at the single marker level may not be able to address the underlying biological interactions that contribute to disease mechanism and may contribute to the issue of “missing heritability.” We performed a genome-wide association study (GWAS) and a genome-wide interaction study (GWIS) of an amyloid imaging phenotype, using the data from Alzheimer's Disease Neuroimaging Initiative. We investigated the genetic main effects and interaction effects on cingulate amyloid-beta (A*β*) load in an effort to better understand the genetic etiology of A*β* deposition that is a widely studied AD biomarker. PLINK was used in the single marker GWAS, and INTERSNP was used to perform the two-marker GWIS, focusing only on SNPs with *p* ≤ 0.01 for the GWAS analysis. Age, sex, and diagnosis were used as covariates in both analyses. Corrected *p* values using the Bonferroni method were reported. The GWAS analysis revealed significant hits within or proximal to *APOE*, *APOC1*, and *TOMM40* genes, which were previously implicated in AD. The GWIS analysis yielded 8 novel SNP-SNP interaction findings that warrant replication and further investigation.

## 1. Introduction

Alzheimer's disease (AD) is the most common neurodegenerative disorder characterized by a progressive decline in memory and cognition. The pathologic cascade in AD involves two primary hallmarks: amyloid-*β* (A*β*) plaques and neurofibrillary tangles [1]. Genetics plays an important role in late-onset Alzheimer's disease (LOAD), but missing heritability remains to be found according to current approximations [2]. The last several decades of research yielded only one genetic risk factor of large effect for LOAD: Apolipoprotein E (*APOE*) with 2 copies of the *ε*4 allele confers approximately 6- to 30-fold risk for the disease [3]. Some recent genome-wide association studies (GWAS) have identified several additional AD susceptibility genes, including* BIN1*,* CLU*,* ABCA7*,* CR1*,* PICALM*,* MS4A6A*,* MS4A4E*,* CD33*,* CD2AP*, and* EPHA1* [4–9]. However, these genetic factors have relatively low effect sizes (odds ratios of 0.87–1.23) and cumulatively account for approximately 35% of population-attributable risk [8]. More recently, a large scale GWAS meta-analysis identified 11 new susceptibility loci with also small effect sizes [10].

Traditional GWAS analyses used discrete disease status as the phenotypic trait of interest despite the fact that LOAD is a clinically heterogeneous disorder. Recently, researchers started to explore intermediate quantitative traits (QTs), such as clinical or cognitive features, biochemical assays, or neuroimaging biomarkers, in genetic association testing. This may have the potential to address the issue of clinical heterogeneity in LOAD. These QTs are often measured as continuous variables and thus exhibit a higher genetic signal-to-noise ratio. Further, most intermediate QTs are more proximal to their genetic bases than disease status. Thus, the incorporation of intermediate QTs can potentially increase statistical power to detect disease-related genetic associations [11, 12]. An ancillary benefit of using QTs is that they can serve as effective biomarkers for monitoring disease progress or treatment response in clinical practice or drug trials.

Over the past 10–15 years, studies have identified robust and predictive biomarkers for AD including levels of tau and amyloid-*β* peptides in cerebrospinal fluid (CSF), selective measures of brain atrophy using magnetic resonance imaging (MRI), and imaging of glucose hypometabolism and amyloid using positron emission tomography (PET) [13]. PET imaging can be used to quantify levels of amyloid in the brain by utilizing a radiotracer such as florbetapir (^18^F-AV-45 or AV-45) or/and Pittsburgh compound-B (PiB, N-methyl-[^11^C]^2^-(40-methylaminophenyl)-6-hydroxybenzothiazole). These amyloid measures have been studied as biomarkers for classifying AD [14–17]. All these multimodal biomarkers can potentially be served as AD relevant QTs and have been examined in many existing quantitative genetics studies of LOAD [18].

In addition, most genetic association studies compute statistics at the single marker level and ignore the possible underlying biological interactions that contribute to the development of disease [19] and could be a possible source for “missing heritability.” Given the quadratically growing search space of two-way interactions, we are facing major computational and statistical challenges. To address this issue, one approach is to effectively explore epistatic interactions in genome-wide data by using a priori statistical and/or biological evidence to generate a reduced set of genetic markers for interaction testing. Using this strategy, previous interaction studies in LOAD (e.g., [20–24]) implicated interactions between* CR1* and* APOE* using quantified A*β* PET as the outcome variable [24] and between cholesterol trafficking genes [21, 22] and tau phosphorylation genes [20] in case-control analyses. These studies demonstrated that the important information could be garnered from investigating genetic interactions in complex diseases like LOAD.

With these observations, in the present work, we conducted a quantitative genetics study of an AD-associated amyloid imaging phenotype and examined both single marker main effects and two-marker interaction effects at the genome-wide level. Specifically, we investigated the main and interaction effects of genome-wide markers on cingulate amyloid-beta (A*β*) load in an effort to better understand the genetic etiology of cingulate cortical A*β* deposition (a LOAD biomarker).

## 2. Materials and Methods

Data used in the preparation of this paper were obtained from the Alzheimer's Disease Neuroimaging Initiative (ADNI) database (http://adni.loni.usc.edu/). The ADNI was launched in 2003 by the National Institute on Aging (NIA), the National Institute of Biomedical Imaging and Bioengineering (NIBIB), the Food and Drug Administration (FDA), private pharmaceutical companies, and nonprofit organizations, as a $60 million, 5-year public-private partnership. The primary goal of ADNI has been to test whether serial magnetic resonance imaging (MRI), positron emission tomography (PET), other biological markers, and clinical and neuropsychological assessment can be combined to measure the progression of mild cognitive impairment (MCI) and early Alzheimer's disease (AD). Determination of sensitive and specific markers of very early AD progression is intended to aid researchers and clinicians to develop new treatments and monitor their effectiveness, as well as lessen the time and cost of clinical trials.

The Principal Investigator of this initiative is Michael W. Weiner, M.D., VA Medical Center and University of California, San Francisco. ADNI is the result of efforts of many coinvestigators from a broad range of academic institutions and private corporations, and subjects have been recruited from over 50 sites across the US and Canada. The initial goal of ADNI was to recruit 800 subjects but ADNI has been followed by ADNI-GO and ADNI-2. To date these three protocols have recruited over 1500 adults, aged 55 to 90, to participate in the research, consisting of cognitively normal older individuals, people with early or late MCI, and people with early AD. The follow-up duration of each group is specified in the protocols for ADNI-1, ADNI-2, and ADNI-GO. Subjects originally recruited for ADNI-1 and ADNI-GO had the option to be followed in ADNI-2. For up-to-date information, see http://www.adni-info.org/.

We applied for and were granted permission to use data from the ADNI cohort (http://www.adni-info.org/) to conduct genetic association and interaction analyses.

### 2.1. Subjects and Data

For the present work, analyses were restricted to subjects with both genotyping data and AV-45 PET data available. The study sample (*N* = 602) included 190 healthy control (HC), 215 early MCI (EMCI), 152 late MCI (LMCI), and 45 AD subjects.  Table 1 shows selected demographic and clinical characteristics of these participants at the time of the baseline AV-45 PET scan.

### 2.2. Genotyping Data and Quality Control

The genotyping data of the participants were collected using either the Illumina 2.5 M array (a byproduct of the ADNI whole genome sequencing sample) or the Illumina OmniQuad array [18, 25, 26]. For the present analyses, we included single nucleotide polymorphism (SNP) markers that were present on both arrays.

Quality control (QC) was performed using the PLINK software (version 1.07) [27]. SNPs not meeting any of the following criteria were excluded from further analyses: (1) call rate per SNP ≥95%; (2) minor allele frequency ≥ 5% (*n* = 117, 175 SNPs were excluded based on criteria 1 and 2); and (3) Hardy-Weinberg equilibrium test of *p* ≥ 10^−6^ (*n* = 997 SNPs were excluded) using control subjects only. Participants were excluded from the analysis if any of the following criteria were not satisfied: (1) call rate per participant ≥ 90% (3 participants were excluded); (2) sex check (1 participant was excluded); and (3) identity check for related pairs (3 sibling pairs were identified with PI_HAT >0.5; one participant of each pair was randomly selected and excluded from the study).

Population stratification analysis was performed using EIGENSTRAT [28] and confirmed using STRUCTURE [29]. It yielded 47 study participants who did not cluster with the remaining subjects and with the CEU HapMap samples who are primarily of European ancestry (non-Hispanic Caucasians). These 47 participants were excluded from the analysis. After QC, 582,718 SNPs and 602 samples remained available for genetic association and interaction analyses.

### 2.3. Quantitative Traits

A previous AV-45 PET study [30] showed that both AD and amnestic MCI subjects had higher standardized uptake value ratio (SUVR) in global cortical, precuneus, frontal, occipital, and posterior cingulate areas. We focused this study in one of these regions, which is cingulate. UC Berkeley extracted baseline SUVR mean measure from the cingulate cortical region (version 2014.7.30) that was downloaded from the ADNI database (http://adni.loni.usc.edu/) for 987 ADNI-GO/2 participants. We also downloaded the cerebellum SUVR measure and used it to normalize the cingulate SUVR measure. The normalized SUVR was used as the quantitative trait (QT) in our analyses. After excluding 383 participants due to the lack of genotyping data, 602 individuals remained in the further analysis.

In addition, amyloid-*β* 1-42 peptide (A*β*-42), total tau (t-tau), and tau phosphorylated at the threonine 181 (p-tau181p), measured in CSF samples, are potential diagnostic biomarkers for AD [31–33]. Among the 602 individuals, 504 have both AV-45 data and CSF data. Following a previous GWAS study on CSF biomarkers [34], QC was performed on the CSF data to reduce the potential influence of extreme outliers on statistical results. Mean and standard deviation (SD) of Aß1-42 and 2 ratios (t-tau/Aß1-42 and p-tau181p/Aß1-42) were calculated, blind to diagnostic information. Subjects who had at least one value greater or smaller than 4 SDs from the mean value of each of 3 CSF variables were regarded as extreme outliers and removed from the analysis. This step removed 5 additional participants, resulting in 499 valid CSF samples.

### 2.4. Genetic Association Studies: Main Effects and Interaction Effects

For GWAS examining the main effects, linear regression was performed using PLINK to determine the association of each SNP to the AV-45 measure. An additive genetic model was tested with covariates including age, gender, and diagnosis (through four binary dummy variables indicating HC, EMCI, LMCI, or AD). Manhattan plots and Q-Q plots were generated using Haploview (http://www.broad.mit.edu/mpg/haploview/) and R (http://www.r-project.org/), respectively.

For GWIS examining the interaction effects, the INTERSNP software [35] was applied to the genotyping data and phenotypic AV-45 measure. First, a single marker *p* value for the main effect was computed for each SNP. Top 10,000 SNPs with the smallest *p* values were selected and included in the subsequent interaction analysis. An explicit test for additive interaction (the full model including both additive and dominance effects plus interaction term versus reduced model that does not contain interaction terms) was performed on all possible SNP pairs among the top 10,000 SNPs, using two-marker analysis. The computation was conducted in a linear regression framework. We examined the association between SNP-SNP interactions and the AV-45 measure while controlling for relevant covariates at the baseline scan, including age, sex, and clinical diagnosis. This resulted in a total of approximately 50 million unique SNP pairs to be tested from the ADNI dataset. Interactions were considered significant if their Bonferroni corrected *p* value < 0.05.

### 2.5. Post Hoc Analysis

For identified significant interactions, we applied hierarchical linear regression using IBM SPSS 20 to estimate the amount of variance (*R* ^2^) in the AV-45 measure accounted for by these interaction terms. We first included the same set of covariates (age, gender, and diagnosis) in the linear model. After that, we included* APOE* status, the closest SNP to the* BCHE* SNP identified in a prior amyloid GWAS study [36], and the two SNP main effects from the identified SNP pair. Finally, we included the SNP-SNP interaction term to calculate additional variance explained by the interaction term. The difference in *R* ^2^ for the significant models was calculated in SPSS as Δ*R* ^2^ = *R* ^2^ (full model with interaction term) − *R* ^2^ (reduced model without interaction term). Significant effects were plotted in SPSS as well.

In addition, based on the identified interactions associated with AV-45, we further evaluated their main and interaction effects on the CSF levels related to amyloid, including A*β*1-42, t-tau181p/A*β*1-42, and p-tau/A*β*1-42. These three CSF measures were used as the QTs in 3 separate genetic analyses, following the same method and steps for analyzing AV-45 phenotype as described above.

## 3. Results and Discussion

### 3.1. GWAS Results

**Figure 1:**
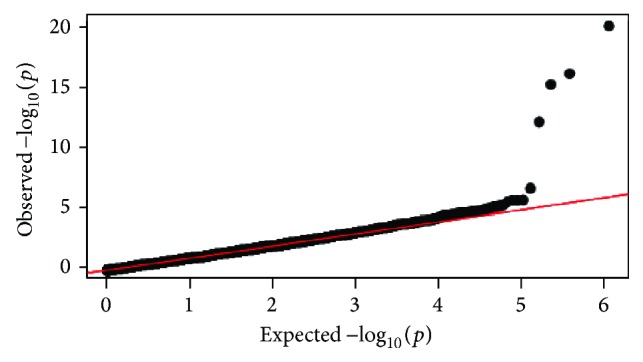
Quantile-quantile (Q-Q) plot of the observed −log⁡_10_⁡*p* values from the GWAS of cingulate cortical Aß load versus those expected under the null hypothesis.

**Figure 2:**
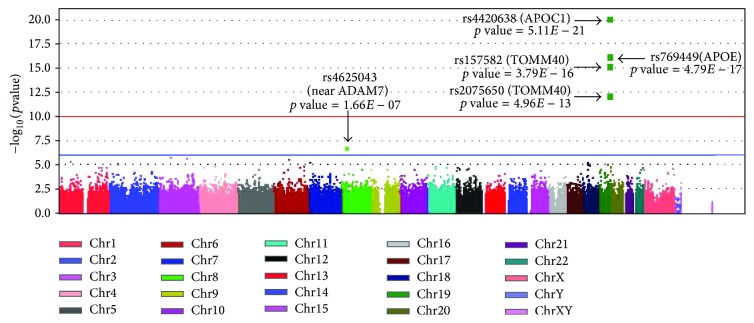
Manhattan plot of the observed −log⁡_10_⁡*p* values from the GWAS of cingulate cortical A*β* load. More than 580,000 SNPs were tested for association with cingulate cortical A*β* burden under an additive model, with age, gender, and diagnosis as covariates. Genome-wide significant associations (exceeding the threshold represented by the red line and determined by Bonferroni correction) were identified on chromosome 19 within the* APOE* and its neighboring regions.

Table 1 shows selected demographic and clinical characteristics of 602 ADNI participants analyzed in this study, where the EMCI group is slightly younger than the other groups.  Figure 1 shows the Q-Q plot, indicating no evidence of spurious inflation.  Figure 2 shows the Manhattan plot. As expected, significant associations were identified between loci on chromosome 19 and the AV-45 measure. The top association is from rs4420638 (*P* = 5.11 × 10^−21^), which codes for the* APOC1* [37]. A few other SNPs within the* APOE* region, including adjacent* APOC1* and* TOMM40*, were significantly associated with the AV-45 level in cingulate.

### 3.2. SNP-SNP Interaction Results

The INTERSNP model we tested included age, sex, and diagnosis as covariates. Eight SNP pairs showed significant interaction effects on the cingulate AV45 measure (corrected *p* value < 0.05) (Table 2): rs2194938 (*CLSTN2*)-rs7644138 (*FHIT*), rs7916162 (*TACC2*)-rs2326536 (*PRNP* ^∗^), rs2295873 (*TACC2*)-rs7794838 (*IGFBP*3^∗^), rs2295874 (*TACC2*)-rs2326536 (*PRNP* ^∗^), rs13056151 (*BCR*)-rs17594541 (*MAGI2*), rs13426621 (LOC388942)-rs7037332 (*TYRP*1^∗^), rs16936424 (LOC387761)-rs10504164 (*N/A*), and rs16939265 (*HNF*4*G* ^∗^)-rs6854047 (*RWDD*4^∗^).

### 3.3. Post Hoc Analysis

Table 2 also shows the results of post hoc analysis on cingulate amyloid deposition. Age, gender, and diagnosis were first included in the model and accounted for 11% of variance in the amyloid QT.* APOE* status was then accounted for an additional 16.1% of variance, followed by the closest SNP to the* BCHE* SNP identified in [36] accounted for an additional 1.8% of variance. For each interaction, we ran a hierarchical linear regression model. We first added in the genetic main effects and then the genetic interaction term to determine the variance associated with the interaction term alone. For rs2194938 (*CLSTN2*)-rs7644138 (*FHIT*), the SNP main effects accounted for 3.4% of variance, and the interaction term accounted for 4.9% of variance (8.3% combined). For rs7916162 (*TACC2*)-rs2326536 (*PRNP* ^∗^), the main effects accounted for 2% of variance, and the interaction accounted for 4.9% of variance (6.9% combined). For rs2295873 (*TACC2*)-rs7794838 (*IGFBP*3^∗^), the main effects accounted for 3.7% of variance, and the interaction term accounted for 4.1% of variance (7.8% combined). For rs2295874 (*TACC2*)-rs2326536 (*PRNP* ^∗^), the SNP main effects accounted for 3.7% of variance, and the interaction term accounted for 4.1% of variance (7.8% combined). For rs13056151 (*BCR*)-rs17594541 (*MAGI2*), the main effects accounted for 3.5% of variance, and the interaction term accounted for 2.6% of variance (6.1% combined). For rs13426621 (LOC388942)-rs7037332 (*TYRP*1^∗^), the main effects accounted for 4.2% of variance, and the interaction accounted for 2.3% of variance (6.5% combined). For rs16936424 (LOC387761)-rs10504164 (*N/A*), the main effects accounted for 3.7% of variance, and the interaction term accounted for 1.7% of variance (5.4% combined). For rs16939265 (HNF4G^∗^)-rs6854047 (RWDD4^∗^), the main effects accounted for 2.7% of variance, and the interaction term accounted for 1.3% of variance (4.0% combined).

Using a slightly reduced sample (*N* = 499) with CSF biomarker data available, all 8 identified interactions remained statistically significant when performing hierarchical linear regression using the CSF phenotypes (one baseline measure: A*β*, two ratios: t-Tau/A*β* and p-Tau/A*β*) instead of the AV-45 measure as outlined earlier (Table 3). We also repeated the same AV-45 analysis on the reduced sample and achieved a very similar result (Table 4).

### 3.4. Discussion

In this study, we performed both GWAS and GWIS analyses of the cingulate AV-45 florbetapir PET measure, using a sample of 602 subjects from the ADNI database. To our knowledge, this is the first genome-wide study on examining SNP-SNP interaction effects on cingulate amyloid deposition in a substantially large sample. In the single marker analysis, as expected, SNPs in* APOE*,* APOC1,* and* TOMM40* genes (Figure 2) exhibited genome-wide significant associations to the cingulate cortical Aß level. Two-marker interaction analyses revealed 8 SNP pairs, which had significant genetic interactions (corrected *p* ≤ 0.05) with cingulate amyloid burden. The risk variants at these pairs had low main effects but explained a relatively high-level variance of the amyloid deposition in cingulate (Table 2).

In addition, missing heritability can partially be explained by the interaction effects that are not examined in traditional GWAS analyses. Genetic risk underlying diagnosis of LOAD is considered to be manifested from multiple genes which interact with each other. We have performed a post hoc analysis investigating the effects of the identified SNP-SNP interactions LOAD related quantitative phenotypes including amyloid deposition and CSF biomarkers (A*β*, t-tau/A*β*, p-tau/A*β*). Given amyloid and tau phosphorylation as major AD hallmarks, it is not surprising to observe the genetic interaction effects on both the amyloid load and relevant CSF biomarkers (Tables 2–4). Our results suggest that significant SNP-SNP interactions could exist between SNPs with low and insignificant main effects, and these interactions could be associated with altered amyloid burden and explain high-level risk in AD.

In line with our hypothesis, we identified multiple significant genetic interactions associated with cingulate amyloid deposition. Several genes found in this study have already been implicated in AD, thus lending confidence to the analytic procedure and results. These genes include* PRNP* [38, 39],* IGFBP3* [40, 41], and* MAGI2* [42, 43]. For example, Guerreiro et al. reported a nonsense mutation in* PRNP* associated with clinical Alzheimer's disease [38]. Ikonen et al. showed that interaction between the Alzheimer's survival peptide humanin and insulin-like growth factor-binding protein 3 (*IGFBP3*) regulates cell survival and apoptosis [40]. Potkin et al. identified an* MAGI2* SNP associated with hippocampal atrophy using the ADNI data [42]. Perhaps more importantly, this study also identified a number of SNPs that had not yet been associated with AD in conventional GWAS studies. Thus, this study exposes several potential candidate genes that could be explored in future replication samples.

This study had several methodological and technical advantages over other imaging genetics studies in addition to the above interesting findings. (1) To our knowledge this is the first genome-wide study to explore how SNP-SNP interactions influence cingulate amyloid burden, measured using florbetapir PET scan information. (2) Using continuous quantitative traits as phenotypes confers higher statistical power than using conventional clinical status. (3) The sample in this study included HC, EMCI, LMCI, and AD, thus providing a continuous and wide spectrum of the disease progression in the dataset. (4) Our approach embraced, rather than ignored, the confounding factors including age, sex, diagnosis, and previously identified risk genes* APOE* and* BCHE* and provided more accurate estimate of the interaction effects on amyloid burden. (5) CSF data were used in this study to cross-check the identified interactions, which had the potential to serve as an indirect validation strategy or provide complemental information.

Our study has several limitations. (1) We used single marker main effect value to select SNPs for interaction analysis, which could miss significant interactions between SNPs with insignificant main effects. (2) The small cell size in the interaction analyses might introduce false positives. (3) Our approach is mostly data-driven, without utilizing any existing biological knowledge (e.g., pathways, networks, and other functional annotation data), which may reduce the statistical power and result interpretability.

## 4. Conclusions

We performed GWAS and GWIS using amyloid imaging as the quantitative phenotype and investigated the genetic interaction effects on cingulate amyloid-beta (A*β*) load. The single marker analyses revealed significant hits within or proximal to* APOE*,* APOC1,* and* TOMM40* genes, which were previously implicated in AD. The interaction analyses yielded a few novel interaction findings associated with cingulate amyloid burden, such as those between* CLSTN2* and* FHIT*, between* TACC2* and* PRNP*, between* TACC2* and* IGFBP3*, and between* BCR* and* MAGI2*. Each of these SNP pairs demonstrated significant interaction effects while their individual main effects were not prominent. This suggests that searching for interaction effects may help solve the problem of missing heritability to some extent. Future studies should attempt to replicate these results in independent datasets with neuroimaging and genetic data, as they become available. Additional pathway analysis and gene sets enrichment analysis could be performed to help understand the genetic interactions between SNPs on amyloid imaging phenotypes and potentially provide critical functional evidence in support of the statistical association findings.

## Figures and Tables

**Table 1:** Selected demographic and clinical characteristics of participants at the time of AV-45 PET scan.

	HC (*N* = 190)	EMCI (*N* = 215)	LMCI (*N* = 152)	AD (*N* = 45)
Age (years)	74.51 (5.74)	71.43 (7.28)	73.03 (7.49)	74.87 (9.05)
Women	94 (49%)	95 (44%)	62 (41%)	17 (38%)
Education (years)	16.53 (2.64)	15.95 (2.66)	16.32 (2.90)	15.67 (2.70)
*APOE* e4 allele present	54 (28%)	87 (40%)	78 (51%)	33 (73%)
CDR-SOB	0.03 (0.13)	1.22 (0.72)	1.73 (0.94)	4.36 (1.64)
Mini mental status examination	29.07 (1.20)	28.39 (1.46)	27.25 (1.77)	22.93 (2.08)
Logical memory immediate recall (WMS-R)	14.46 (3.08)	10.96 (2.77)	7.32 (3.06)	4.40 (2.52)
Logical memory delayed recall (WMS-R)	13.55 (3.27)	8.90 (1.72)	4.22 (2.75)	2.02 (2.17)

Normalized SUVR of cingulate amyloid burden	1.211 (0.21)	1.273 (0.23)	1.274 (0.27)	1.48 (0.24)

AD: Alzheimer's disease; CDR–SOB: clinical dementia rating-sum of boxes; EMCI: early mild cognitive impairment; HC: healthy control; LMCI: late mild cognitive impairment; PET: positron emission tomography; WMS-R: Wechsler Memory Scale-Revised. Data are shown in the format of “number (%)” or “mean (SD).”

**Table 2:** Results of sample (*N* = 602): eight SNP-SNP interactions associated with cingulate amyloid burden. The Bonferroni corrected *p* values (<0.05) and *R* ^2^ of the SNP-SNP interaction term are shown in bold.

Number	SNP1 × SNP2	Gene	CHR	Main effect	Interaction		*R* square				
				*p* value	*p* value	Corrected *p* value	Age + Sex + Dx^a^	*APOE* ^b^	*BCHE* ^c^	SNP1 + SNP2^d^	SNP1 ∗ SNP2^e^
1	rs2194938 × rs7644138	CLSTN2	3	0.000481499	5.24*E* − 10	**0.026**	0.110	0.161	0.018	0.034	**0.049**
		FHIT	3	0.000993424							

2	rs7916162 × rs2326536	TACC2	10	0.00897357	7.81*E* − 10	**0.038**	0.110	0.161	0.018	0.020	**0.049**
		PRNP^∗^	20	0.00850742							

3	rs2295873 × rs7794838	TACC2	10	0.000291361	7.01*E* − 10	**0.035**	0.110	0.161	0.018	0.037	**0.041**
		IGFBP3^∗^	7	0.00973379							

4	rs2295874 × rs2326536	TACC2	10	0.0016572	8.62*E* − 10	**0.042**	0.110	0.161	0.018	0.024	**0.039**
		PRNP^∗^	20	0.00850742							

5	rs13056151 × rs17594541	BCR	22	0.0015002	9.86*E* − 10	**0.048**	0.110	0.161	0.018	0.035	**0.026**
		MAGI2	7	0.00360174							

6	rs13426621 × rs7037332	LOC388942	2	6.72*E* − 06	3.44*E* − 10	**0.017**	0.110	0.161	0.018	0.042	**0.023**
		TYRP1^∗^	9	0.00386214							

7	rs16936424 × rs10504164	LOC387761	11	1.24*E* − 05	9.22*E* − 10	**0.045**	0.110	0.161	0.018	0.037	**0.017**
		NA	8	0.00166034							

8	rs16939265 × rs6854047	HNF4G^∗^	8	0.000407669	6.95*E* − 10	**0.034**	0.110	0.161	0.018	0.027	**0.013**
		RWDD4^∗^	4	0.000464343							

^a^Age + Sex + Dx: percent of variance in cingulate amyloid burden explained by age, gender, and diagnosis.

^b^ *APOE*: percent of additional variance in cingulate amyloid burden explained by the APOE genotype after accounting for age, gender, and diagnosis.

^c^ *BCHE*: percent of additional variance in cingulate amyloid burden explained by the *BCHE* SNP after accounting for age, gender, diagnosis, and *APOE* genotype.

^d^SNP1 + SNP2: percent of additional variance in cingulate amyloid burden explained by the combined main effect of SNP1 and SNP2 after accounting for age, gender, diagnosis, *APOE* genotype, and the *BCHE* SNP.

^e^SNP1 ∗ SNP2: percent of additional variance in cingulate amyloid burden explained by the interaction effect of SNP1 and SNP2 after accounting for age, gender, diagnosis, *APOE* genotype, the *BCHE* SNP, SNP1, and SNP2.

^∗^Nearest gene proximal to the SNP.

**Table 3:** Results of sample (*N* = 499): eight SNP-SNP interaction associations with three CSF biomarkers.

Number	SNP1 × SNP2	A*β* (*R* square)					t-Tau/A*β* (*R* square)					p-Tau/A*β* (*R* square)				
		Age + Sex + Dx	APOE	BCHE	SNP1 + SNP2	SNP1 ∗ SNP2	Age + Sex + Dx	APOE	BCHE	SNP1 + SNP2	SNP1 ∗ SNP2	Age + Sex + Dx	APOE	BCHE	SNP1 + SNP2	SNP1 ∗ SNP2
1	rs2194938 × rs7644138	0.108	0.187	0.012	0.021	0.008	0.132	0.153	0.022	0.016	0.014	0.134	0.129	0.024	0.007	0.011

2	rs7916162 × rs2326536	0.108	0.187	0.012	0.003	0.019	0.132	0.153	0.022	0.004	0.011	0.134	0.129	0.024	0.005	0.014

3	rs2295873 × rs7794838	0.108	0.187	0.012	0.014	0.012	0.132	0.153	0.022	0.017	0.011	0.134	0.129	0.024	0.016	0.007

4	rs2295874 × rs2326536	0.108	0.187	0.012	0.003	0.018	0.132	0.153	0.022	0.002	0.012	0.134	0.129	0.024	0.003	0.015

5	rs13056151 × rs17594541	0.108	0.187	0.012	0.005	0.005	0.132	0.153	0.022	0.005	0.006	0.134	0.129	0.024	0.005	0.004

6	rs13426621 × rs7037332	0.108	0.187	0.012	0.002	0.002	0.132	0.153	0.022	0.006	0.012	0.134	0.129	0.024	0.004	0.006

7	rs16936424 × rs10504164	0.108	0.187	0.012	0.018	0.004	0.132	0.153	0.022	0.014	0.008	0.134	0.129	0.024	0.009	0.003

8	rs16939265 × rs6854047	0.108	0.187	0.012	0.012	0.007	0.132	0.153	0.022	0.003	0.005	0.134	0.129	0.024	0.002	0.003

**Table 4:** Results of sample (*N* = 499): eight SNP-SNP interactions associated with cingulate amyloid burden.

Number	SNP1 × SNP2	Gene	CHR	Main effect	Interaction		*R* square				
				*p* value	*p* value	corrected *p* value	Age + Sex + Dx	APOE	BCHE	SNP1 + SNP2	SNP1 ∗ SNP2
1	rs2194938 × rs7644138	CLSTN2	3	0.000481499	5.24*E* − 10	**0.026**	0.127	0.133	0.019	0.047	**0.053**
		FHIT	3	0.000993424							

2	rs7916162 × rs2326536	TACC2	10	0.00897357	7.81*E* − 10	**0.038**	0.127	0.133	0.019	0.027	**0.055**
		PRNP^∗^	20	0.00850742							

3	rs2295873 × rs7794838	TACC2	10	0.000291361	7.01*E* − 10	**0.035**	0.127	0.133	0.019	0.059	**0.059**
		IGFBP3^∗^	7	0.00973379							

4	rs2295874 × rs2326536	TACC2	10	0.0016572	8.62*E* − 10	**0.042**	0.127	0.133	0.019	0.031	**0.046**
		PRNP^∗^	20	0.00850742							

5	rs13056151 × rs17594541	BCR	22	0.0015002	9.86*E* − 10	**0.048**	0.127	0.133	0.019	0.034	**0.037**
		MAGI2	7	0.00360174							

6	rs13426621 × rs7037332	LOC388942	2	6.72*E* − 06	3.44*E* − 10	**0.017**	0.127	0.133	0.019	0.049	**0.039**
		TYRP1^∗^	9	0.00386214							

7	rs16936424 × rs10504164	LOC387761	11	1.24*E* − 05	9.22*E* − 10	**0.045**	0.127	0.133	0.019	0.051	**0.023**
		NA	8	0.0166034							

8	rs16939265 × rs6854047	HNF4G^∗^	8	0.000407669	6.95*E* − 10	**0.034**	0.127	0.133	0.019	0.040	**0.026**
		RWDD4^∗^	4	0.000464343							

^∗^Nearest gene proximal to the SNP.
